# Cumulative impact of cover crops on soil carbon sequestration and profitability in a temperate humid climate

**DOI:** 10.1038/s41598-020-70224-6

**Published:** 2020-08-07

**Authors:** Inderjot Chahal, Richard J. Vyn, Danielle Mayers, Laura L. Van Eerd

**Affiliations:** 1 School of Environmental Sciences, University of Guelph, Ridgetown Campus, Ridgetown, Canada; 2 Department of Food, Agricultural, and Resource Economics, University of Guelph, Ridgetown Campus, Ridgetown, Canada; 3 Department of Food, Agricultural, and Resource Economics, University of Guelph, Guelph, Canada

**Keywords:** Biogeochemistry, Environmental sciences, Environmental social sciences

## Abstract

Although soil C sequestration with cover crops (CCs) has been linked with the potential of CCs in climate change mitigation, the long-term usage of CCs on soil C storage and farm-based economics have been widely overlooked. Therefore, in a CC experiment established in 2007 in a temperate humid climate, four CCs and a no-CC control were compared to evaluate their potential to sequester C and provide economic returns. Total amount of plant C added to soil with CCs translated into greater soil organic carbon (SOC) content by 10–20 Mg C ha^−1^ than the no-CC control across both sites. Greater crop yield and reduced yield variability with CCs suggest the long-term potential of CCs in increasing agroecosystem resiliency. Moreover, greater profit margins with CCs in processing vegetable crops but not grain and oilseed crops indicate CC effects on crop profitability are dependent on the production system. Our study results indicated that the loss in profit margins with CC usage in grain and oilseed crops might be overcome with C pricing (at $50 Mg^−1^) on quantity of C sequestered after 9 years of CCing; thus, providing financial compensation to growers may be a mechanism to encourage CC adoption.

## Introduction

Climate change, due to an increase in the greenhouse gas (CO_2_, CH_4_, and N_2_O) emissions, is considered one of the most important environmental challenges of the present century^1^. Storing atmospheric CO_2_ in the soil is a key strategy for mitigating climate change^2,3^ due to a greater potential of soil to store C than atmosphere^4^. Globally, soil C pool is 3.2 times bigger than the atmospheric pool and 4 times than the biotic pool^5,6^. However, ability of soil to sequester atmospheric C, predominantly as soil organic carbon (SOC), varies across land management practices, crop rotations, quantity of C inputs, climatic conditions, and soil texture.

In addition to C sequestration, C trading has gained attention globally. Carbon trading refers to market-based financial incentives provided to practitioners for controlling greenhouse gas emissions and/or sequestering C^7^. Incentivising C storage enhances the economic value of the soil ecosystem services of SOC provided by the agricultural management practices^8,9^, and providing payments contributes to the financial well-being of the growers who invest in C sequestration strategies. There is the potential for producers to benefit financially from the additional C sequestration in Canada through a C pricing policy that was recently implemented as a component of the Pan-Canadian Framework on Clean Growth and Climate Change^10^. Under this framework, the C price was $20 per tonne in 2019 and is set to rise by $10 per year to $50 per tonne in 2022; hence clear C pricing has been actualized in Canada. Therefore, a mechanism that enables producers to benefit financially from increasing C sequestration through specific production practices, either by increasing profits or by offsetting the additional costs, is needed.

A major pathway to build soil C storage is by increasing the quantity of C inputs. Adoption of cover crops (CC) in the cropping system is recommended as a management strategy for increasing SOC stores^11^. In addition to providing C inputs, CCs offer numerous agroecosystem services such as reduce N losses via leaching^12^, increase yield of subsequent crop^13^, and improve overall soil quality^13,14^. Despite the aforementioned benefits, CC-induced effects on soil and crop productivity are highly dependent on CC management practices (time of CC planting and termination, CC species, and cropping system, duration of the experiment), quantity and quality of CC residues, and the climatic conditions^15^. Therefore, assessment of SOC storage in response to CC treatments is needed for various production systems.

Cover crop research has evaluated CC effects on soil quality^13–14,16–17^ and crop production^16,18–22^. Long-term studies are needed to assess the potential of CCs to sequester C^4,23–25^ and have primarily focused on evaluating the amount of soil C sequestered with CCs than control^26–28^ rather than the change in SOC stock^29^. Consistent with aforementioned long-term studies, we evaluated the differences in surface SOC storage with and without CCs in our experiment. Soil organic C represents a stable pool of soil C, which is rarely impacted by the seasonal variation due to climatic conditions^30^. It takes several years (≥ 6-years) to detect changes in SOC stocks due to CCs^23,29^ and therefore, long-term studies are needed to provide a quantitative evidence about CC effects on SOC storage. The results from the long-term trials assist growers in making improved decisions about selecting appropriate CCs for enhancing agroecosystem services, primary productivity and profit margins.

Evidence in the literature regarding the impacts of CCs on crop yields is inconclusive, as there is considerable variability in results across studies. For example, while some studies have found that CCs positively affect subsequent crop yields^31,32^, other studies have found no significant effect^33,34^ or even decreased yields^35,36^. There is even less evidence regarding impacts of CCs on profit margins. For example, Schomberg et al*.*^37^ reported that returns for cotton in the southern USA were higher with winter CCs, while Flower et al*.*^38^ observed lower profit margins with CCs in a cereal rotation in Australia. Due to the lack of studies discussing CC impacts on profit margins, two recent studies have highlighted the need for additional research on the long-term economic returns from CCs^23,39^.

The objectives of this study were to (i) evaluate the cumulative effects of CC on soil C sequestration, primary productivity, and profit margins, and (ii) determine if current and projected C prices may be an effective strategy for incentivising the quantity of soil C sequestered by CCs. To meet these objectives, a long-term CC experiment located at Ridgetown, Ontario Canada, established in 2007 and repeated at an adjacent site in 2008 was utilized^13–14,18–20,40^. Previously in this experiment, CC treatment effects on N fertility, crop productivity, and soil quality have indicated enhancements with CCs compared to the no cover crop control (no-CC)^13–14,18–20,40^. The Ridgetown experiment is one of the very few longest running CC trials making direct comparisons among CCs in North America. This provides a unique opportunity to advance our understanding of the CC-induced effects on soil C storage across different CC treatments. In addition, by evaluating the economics of CCing combined with C price incentives, insights into CC usage and the potential need for financial compensation may emerge. To our knowledge, this is the first quantification of CC economics that includes pricing for environmental goods and services (C sequestration).

## Results

### Annual and cumulative above-ground C assimilation

**Figure 1:**
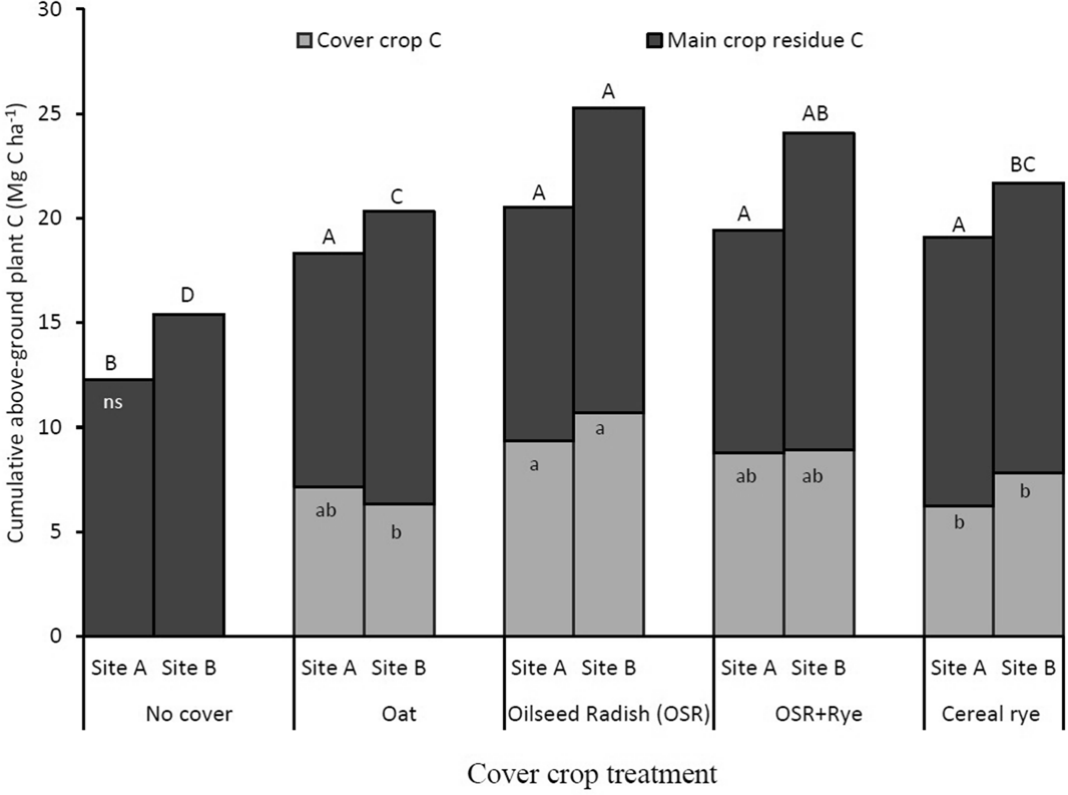
Cumulative above-ground plant C inputs from cover crops (light gray bars) and main crop (dark gray bars) over the period of 9-years at site A and site B at Ridgetown, Ontario. At each site and for each parameter, bars with different letter and capitalization indicate statistical significance at *P* < 0.05 per protected LSD test. Error bars represent standard error of the mean.; ns = not significant (*P* > 0.05).

At both sites, annual and cumulative plant above-ground C contents were calculated and represented the sum of C assimilation from CCs and main crops planted each year and C accrual over the duration of the study, respectively. Differences in C assimilation among years largely reflect the main crop grown and the length of time the cover crop grew as well as weather conditions (Table 1). At both sites, among the tested CCs, OSR (avg. 1.11 Mg C ha^−1^ year^−1^), had the greatest annual above-ground C content (Table 1). Cumulative CC C inputs were 7.87–8.42 Mg C ha^−1^ at the two sites (Fig. 1). But no differences in the main crop C content among the CC treatments were detected at both sites (Fig. 1). Hence over the 9 years, oilseed radish (OSR, avg. 22.8 Mg C ha^−1^) had the greatest cumulative plant (main crop and CC) C while no-CC was the least (avg. 13.8 Mg C ha^−1^, main crop only) at both sites (Fig. 1). Thus, soil C inputs were attributed to CC, rather than main crops.

**Table 1:** Effect of cover crops on annual above-ground CC C content from 2007 to 2015 at site A and 2008 to 2016 at site B at Ridgetown, Ontario.

Cover crop (CC)		Annual above-ground CC C inputs	
		Mg C ha^−1^	
		Site A	Site B
No cover		–^z^	–
Oat		0.795*b*	0.699*d*
Oilseed radish (OSR)		1.04*a*	1.180*a*
OSR + Rye		0.978*a*	0.989*b*
Cereal rye		0.695*b*	0.869*c*
SE^y^		0.030	0.027
Year	Main crop followed by CC		
2007/08	Pea fb^x^ CC	1.94*a*	1.93*a*
2008/09	Sweet corn fb CC	0.716* cd*	1.31*b*
2009/10	Spring wheat fb CC	0.852*bc*	0.892* cd*
2010/11	Tomato fb CC	1.01*b*	0.759*d*
2011/12	Grain corn	–	–
2012/13	Squash fb CC	0.645*d*	0.584*e*
2013/14	Soybean	–	–
2014/15	Winter wheat fb CC	1.02*b*	1.03*c*
2015/16	Tomato fb CC	0.106*e*	0.224*f*
SE		0.046	0.037
Effects		*P values*	
CC		** < 0.0001**	** < 0.0001**
Year		** < 0.0001**	** < 0.0001**
CC × year		** < 0.0001**	** < 0.0001**

^a^^−^^f^In each column and each effect, means followed by a different letter indicate statistical significance at *P* < 0.05 per protected LSD test.

^z^No cover crop was planted in control plots or all plots after grain corn or soybean harvest.

^y^SE = standard error of mean.

^x^fb = followed by.

Bold font indicates statistical significance at *P* < 0.05.

OSR + Rye = mixture of oilseed radish and cereal rye.

### Soil organic C storage

**Figure 2:**
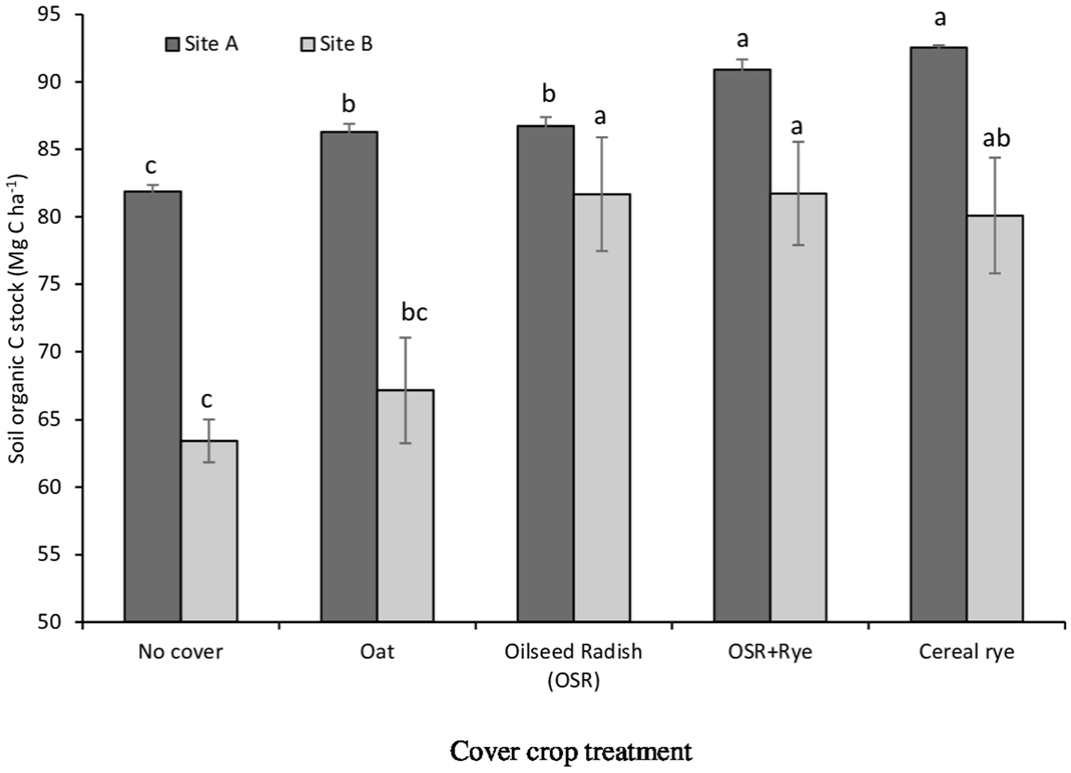
Long-term effect of cover crops on soil organic C stock from 0 to 15 cm depth measured in September 2015 at site A (dark gray bars) and September 2016 at site B (light gray bars) at Ridgetown, Ontario. At each site, bars with a different letter indicate statistical significance at *P* < 0.05 per protected LSD test. Error bars represent standard error of the mean.

The observed differences in C assimilation between CC and the no-CC control translated into differences in surface SOC storage (15 cm) measured in 2015 (site A) and 2016 (site B). Compared with the no-CC (81.8 Mg C ha^−1^ at site A and 63.4 Mg C ha^−1^ at site B), CCs had significantly greater SOC content at both sites (avg. 89.1 Mg C ha^−1^ at site A avg. 77.6 Mg C ha^−1^ at site B, Fig. 2). Trends observed for SOC content were cereal rye = OSR + Rye > OSR = oat > no-CC at site A and OSR + Rye = OSR ≥ cereal rye ≥ oat ≥ no-CC at site B (Fig. 2). Thus, the CCs tested sequestered 4.4–10.6 Mg C ha^−1^ more C than the no-CC control at site A with a greater range of differences between CCs and no-CC control at site B (3.7–18.3 Mg C ha^−1^).

Furthermore, association between average crop yield ratio over the study duration and SOC content, measured from 0 to 15 cm depth, was investigated at both sites. No clear relationship was observed between average crop yield ratio and SOC content at both sites (R^2^ = 0.002, r = 0.04, *P* = 0.7779 at site A; R^2^ = 0.018, r = 0.13, *P* = 0.4091 at site B). A negative but non-significant relationship was observed between average crop yield ratio variability and SOC content at both sites (R^2^ = 0.026, r = − 0.17, *P* = 0.3374 at site A; R^2^ = 0.115, r = − 0.34, *P* = 0.8618 at site B), indicating a potential link between greater SOC content and lesser variability in crop yield.

### Impact of CCs on crop productivity and economics

Pearson correlations between the CC parameters (fall above-ground CC C content) and the subsequent crop yield (main crop in the rotation) revealed that at site A, fall above-ground CC C content (R^2^ = 0.332, r = − 0.57, *P* < 0.0001) correlated negatively with grain corn yield in 2011 and soybean grain yield in 2013 (R^2^ = 0.144, r = − 0.37, *P* = 0.0156; Table 2). However, at site B, out of all the main crops planted in the rotation, only tomato yield positively correlated with fall above-ground CC C content (R^2^ = 0.276, r = 0.52, *P* = 0.0003; Table 2). At both sites, the main crop yield correlated (positively or negatively) with CC parameters when the above-ground CC C content measured in the previous fall was generally low.

**Table 2:** Coefficient of correlation between cover crop above-ground biomass and C content collected in the prior fall and main crop yield for each year at site A and B at Ridgetown, Ontario.

Rotation (main crop yield Mg ha^−1^)	CC above-ground biomass from previous fall (kg ha^−1^)		CC above-ground C content from previous fall (kg C ha^−1^)	
	Site A	Site B	Site A	Site B
Pea	–^y^	–	–	–
CC fb^z^ sweet corn	− 0.065	0.213	− 0.066	0.221
CC fb spring wheat	− 0.157	0.191	− 0.020	0.197
CC fb tomato	− 0.155	0.155	− 0.170	0.049
CC fb grain corn	− **0.485**	0.148	− **0.570**	0.134
Squash	–	–	–	–
CC fb soybean	− **0.388**	0.046	− **0.379**	0.025
Winter wheat	–	–	–	–
CC fb tomato	0.225	**0.594**	0.220	**0.525**

^z^fb = followed by.

^y^No cover crop was planted in the rotation before these main crops (i.e. after grain corn and soybean); therefore, no correlation was evaluated.

Bold font indicates statistically significant correlation at *P* < 0.05.

Long-term impact of CCs was observed on tomato marketable yield at both sites (*P* = 0.0293 at site A in 2015; *P* = 0.0010 at site B in 2016, Table 3). Tomato yield was greatest with oat in 2015 (91.4 ± 3.66 Mg ha^−1^) and OSR in 2016 (124 ± 5.84 Mg ha^−1^, Table 3). However, for the remaining years, crop yield was not different among CC treatments at both sites (Table 3). On average across all crops at both sites, yield ratios for all four CC treatments were significantly greater than no-CC control; though the differences in yield ratio for oat and cereal rye were statistically significant at the 10% level only (Table 4). However, these results were found to vary between grain and oilseed crops and processing vegetable crops. Average yield ratios for the grain and oilseed crops were not significantly different from the no-CC control for any of the four CC treatments. For processing vegetable crops, yields were 8.6–16.3% greater for cereal rye, OSR, and OSR + Rye than the no-CC control. Thus, the impact of CCs on crop productivity depended on the production system (main crop).

**Table 3:** Effect of cover crop on the marketable yield of the main crops from 2008 to 2015 at site A and 2009 to 2016 at site B at Ridgetown, Ontario.

Main crop	Sweet corn		Spring wheat		Tomato		Grain corn		Squash		Soybean		Winter wheat		Tomato	
Site	A	B	A	B	A	B	A	B	A	B	A	B	A	B	A	B
Year	2008	2009	2009	2010	2010	2011	2011	2012	2012	2013	2013	2014	2014	2015	2015	2016
Cover crop	Mg ha^−1^															
No cover	21.1	13.6	2.74	2.15	84.3	95.8	15.2	16.8	34.7	39.3	3.77	3.01	3.99	7.64	79.5*bc*	88.8*b*
Oat	21.5	18.4	3.02	2.59	73.5	105	14.7	17.3	37.9	39.3	3.69	3.16	4.02	7.80	91.4*a*	102*b*
Oilseed Radish (OSR)	21.5	17.9	2.29	2.58	79.3	109	14.6	17.3	41.3	39.2	3.70	3.15	4.21	7.85	85.2*abc*	124*a*
OSR + Rye	22.1	17.1	2.46	2.52	88.0	94.1	15.1	16.9	35.9	39.8	3.57	3.29	4.00	7.53	89.9*ab*	104*ab*
Cereal rye	27.5	18.5	2.03	2.41	77.8	103	14.5	16.9	36.7	40.6	3.65	2.92	4.12	7.03	76.6*c*	102*b*
SE^z^	2.96	1.74	0.238	0.269	8.10	5.36	0.439	0.224	2.80	2.25	0.064	0.153	0.070	0.218	3.66	5.84
Effects	*P values*															
Cover crop	0.5215	0.1687	0.0543	0.5257	0.3043	0.2342	0.2982	0.4399	0.4098	0.9908	0.2240	0.4809	0.1208	0.0518	**0.0293**	**0.0010**

^a^^−^^c^ In each column, means followed by a different letter indicate statistical significance at *P* < 0.05 per protected LSD test.

^z^SE = standard error of mean.

Bold font indicates statistical significance at *P* < 0.05.

OSR + Rye = mixture of oilseed radish and cereal rye.

**Table 4:** Linear mixed regression model parameter estimates (SE) for cover crop effects on yield ratios from 2007 to 2016 (Sites A and B combined) at Ridgetown, Ontario.

Cover crop	All main crops		Grain and oilseed crops		Vegetable crops	
Intercept (no cover crop)	1.0000	***	1.0000	***	1.0000	***
	(0.0266)		(0.0259)		(0.0339)	
Oat	0.0436	*	0.0451		0.0427	
	(0.0235)		(0.0284)		(0.0321)	
Cereal rye	0.0417	*	− 0.0358		0.0860	***
	(0.0235)		(0.0284)		(0.0321)	
Oilseed radish (OSR)	0.1100	***	0.0172		0.1630	***
	(0.0235)		(0.0284)		(0.0321)	
OSR + Rye	0.0864	***	0.0124		0.1287	***
	(0.0235)		(0.0284)		(0.0321)	

*,**, and *** indicates statistical significance at *P* ≤ 0.1, *P* ≤ 0.05, and *P* ≤ 0.01, respectively.

OSR + Rye = mixture of oilseed radish and cereal rye.

Across all crops at both sites (Table 5), the results of regression analysis indicated that profit margin ratios were greater only for OSR and OSR + Rye, relative to the no-CC. Thus, the added costs of CCing were not compensated by the revenue generated by the 4.2–8.6% average yield increases with oat and cereal rye CCs. However, average increase in profit margins of 9.03% with OSR and 5.26% with OSR + Rye were observed across all crops in the rotation. When the additional value of soil C storage was taken into account, based on a carbon price of $20 Mg^−1^, profit margin ratios were once again greater than the no-CC for both OSR and OSR + Rye. At $50 C Mg^−1^, profit margin ratios were also greater for cereal rye. Hence, on average across all crops in the rotation, financial compensation would be required to make CCing profitable for cereal rye and would enhance profitability for OSR and OSR + Rye.

**Table 5:** Linear mixed regression model parameter estimates (SE) for cover crop effects on profit margin ratios across all main crops, grain and oilseed, and vegetable crops from 2007 to 2016 (Sites A and B combined) at Ridgetown, Ontario.

Cover crop	Profit margins without C pricing			Profit margins with C pricing at $20 Mg^−1^			Profit margins with C pricing at $50 Mg^−1^		
	All main crops	Grain and oilseed crops	Vegetable crops	All main crops	Grain and oilseed crops	Vegetable crops	All main crops	Grain and oilseed crops	Vegetable crops
Intercept (no cover crop)	1.0000***	1.0000***	1.0000***	1.0000***	1.0000***	1.0000***	1.0000***	1.0000***	1.0000***
	(0.0274)	(0.0276)	(0.0343)	(0.0279)	(0.0303)	(0.0344)	(0.0293)	(0.0367)	(0.0346)
Oat	0.00280	0.0096	0.0386	0.0331	0.0211	0.0399	0.0406	0.0383	0.0419
	(0.0249)	(0.0321)	(0.0322)	(0.0245)	(0.0319)	(0.0323)	(0.0248)	(0.0347)	(0.0325)
Cereal rye	0.0141	− 0.1035***	0.0813**	0.0315	− 0.0636**	0.0858***	0.0576**	− 0.0037	0.0926***
	(0.0249)	(0.0321)	(0.0322)	(0.0245)	(0.0319)	(0.0323)	(0.0248)	(0.0347)	(0.0325)
Oilseed radish (OSR)	0.0903***	− 0.0348	0.1617***	0.1054***	0.0002	0.1656***	0.1281***	0.0525	0.1713***
	(0.0249)	(0.0321)	(0.0322)	(0.0245)	(0.0319)	(0.0323)	(0.0248)	(0.0347)	(0.0325)
OSR + Rye	0.0526**	− 0.0706**	0.1230***	0.0702***	− 0.0302	0.1275***	0.0965***	0.0303	0.1343***
	(0.0249)	(0.0321)	(0.0322)	(0.0245)	(0.0319)	(0.0323)	(0.0248)	(0.0347)	(0.0325)

*,**, and *** indicates statistical significance at *P* ≤ 0.1, *P* ≤ 0.05, and *P* ≤ 0.01, respectively.

OSR + Rye = mixture of oilseed radish and cereal rye.

As with yield ratios, considerable variation in the results for profit margin ratios was found between grain and oilseed crops and processing vegetable crops. For grain and oilseed crops (Table 5) relative to the no-CC control, profit margin ratios were significantly less for cereal rye and OSR + Rye but not different for oat and OSR. These results changed under scenarios where compensation is received for the value of soil C storage. When compensation was received based on a carbon price of $20 Mg^−1^, the profit margin ratio for OSR + Rye was no longer significantly less than the no-CC control, while at a carbon price of $50 Mg^−1^, profit margin ratios were not different from the no-CC control for any of the CC treatments. Therefore, even with financial compensation for C sequestration in grain and oilseed crops, there were no profit margin gains with CCing.

In contrast to grain and oilseed crops, for processing vegetable crops (Table 5), the profit margin ratios were positive, and analysis results were consistent across the three models. In each case, profit margin ratios were greater for cereal rye, OSR, and OSR + Rye, relative to the no-CC control. The magnitudes of these differences increased as financial compensation for additional soil C sequestration was added. Thus, in the tested vegetable crops, revenue from yield increases exceeded the additional costs of including CCs into the rotation for three of the four CCs tested. Regardless of C payment, profit margins for oat were not different than the no-CC control.

## Discussion

The quantity of C inputs from main crops was not different among CCs relative to CC C assimilation. At both sites, CCs assimilated significant quantities of C. Over the 9-year rotation cumulative aboveground plant C was 12.2–20.2 Mg ha^−1^ at site A and 15.4–25.2 Mg C ha^−1^ at site B. Among the tested CCs, OSR contributed to the greatest annual and cumulative above-ground CC C inputs whereas cereal rye had the least, suggesting the CC effects to be species-specific. Greater C assimilation by OSR is attributed to fast growth and was consistent with greater biomass production. The observed variation in the amount of C assimilated annually by the CCs were primarily related to the main crop grown prior to planting CCs, in addition to differences in CC species and weather variability among years. Similar quantity and variation in CC C content with species and year of sampling were reported by Buchi et al*.*^21^ and Garcia-Gonzalez et al.^41^. These results confirm that the choice of CC species is a critical factor governing the amount of CC biomass and C produced^21^.

Our results confirmed that SOC content was 11–22% greater compared to the no-CC. This amounted to soil C sequestration of 10–20 Mg C ha^−1^ at the two sites. Similar results of greater SOC with CCs than no-CC control has been reported in several studies^11,13,17,23,42–43^ including meta-analyses^29,44–45^ and modelling^16,21,46^ studies. Our results indicated that the above-ground cumulative C additions that contributed to SOC accumulation were largely dominated by CC C inputs rather than main crops. We did not calculate a change in SOC content over the study duration as initial soil C stocks were not quantified; hence, it is not possible to determine if CCs are mitigating C losses or intensifying C stabilization or both. Regardless, in our long-term study, observed greater SOC content with CCs than without indicates the enhanced potential for C storage in production systems with CCs.

The amount of soil C stored in the surface soil over the study duration represented a cumulative effect of CCs. As stated by Poeplau and Don^29^, CC C inputs to the soil undergo various transformations depending on the turnover time of the CC residue in the soil, and a fraction of the added soil C remains in a stable organic form and contributes to SOC accumulation in the long-term. Therefore, the CC-induced effect on soil C storage was assessed by dividing the SOC content measured in 2015 at site A and 2016 at site B, by the number of study years (9-years). Based on a reasonable assumption that pre-treatment SOC content was similar among our plots, the C accumulation rate with CCs was between 1.1 and 2.2 Mg C ha^−1^ year^−1^ in the surface 15 cm. The differences in soil depth largely explains the lower annual C stock change (0.32 Mg ha^−1^) in 0–22 cm soil depth reported via meta-analysis of CC usage^29^. Our results confirm that CC-induced accumulation in SOC content can be substantial; thus, might have long-term implications on mitigating greenhouse gas emissions^21,29,44^ if CC usage were implemented on a large scale.

Although the purpose of this study was not to elucidate the mechanism of CC-induced C sequestration nor to estimate C balance in our production system, it is apparent that above-ground plant C inputs alone do not explain the observed soil C gains. It is not clear if the CCs had greater SOC content by reducing C losses and decomposition of organic matter or by enhancing the protection of soil C in aggregates or due to rhizodeposition. Based on the range of fall CC root to shoot ratios from 0.17 to 1 and root CC C% from 39 to 52%^47–49^, the CCs in our study would have assimilated 0.18–1.74 Mg C ha^−1^ in root biomass. Others agree that below-ground CC biomass has a major influence on increasing SOC stock and fertility than the above-ground biomass production^23,50^. Congruently in our experiment, CCs had greater wet aggregate stability than no-CC by 7.1–8%^14^, which might have contributed to the protection of C from decomposition within aggregates and potentially reduced erosion. Furthermore, CCs had lower water extractable organic C concentration than no-CC (155 mg C kg^−1^ at site A and 436 mg C kg^−1^ at site B) as reported in Chahal and Van Eerd^13^, indicating the potential of CCs in reducing leaching losses of C in our long-term experiment. Irrespective of the mechanism, estimates of C storage to deeper depths are needed to further our understanding on CC C sequestration^29^.

Growers tend to adopt the management practices which tend to increase crop yield and provide economic gains. However, studies evaluating the economic returns from CCs are limited^18–19,51^. Therefore, we assessed the impact on main crop (processing vegetable crops and grain and oilseed crops) yield and profit margins due to CCs in our experiment. In agreement with our results, several other studies have observed equivalent or greater crop yields with CCs^13,17,23,52–54^. A meta-analysis conducted by Norris and Congreves^55^ also reported greater vegetable crop yields and profit margins^18^ with CCs than a control. The mechanism involved in obtaining greater crop yield with CCs than control is not clearly understood but might be attributed to increase in soil fertility and soil quality^17,23,55^ or a decrease in weed pressure^19^. Moreover, the impacts of CCs on profit margins varied considerably between grain and oilseed crops and processing vegetable crops, as CC significantly reduced profit margins for grain and oilseed crops and significantly increased profit margins for vegetable crops. This corresponds with the observed impacts of CCs on yields, where yield ratios for CC treatments were not significantly different from the no-CC control for grain and oilseed crops but were significantly higher for processing vegetable crops. Our results of greater crop yields and profit margins for vegetable crops but not grain and oilseed crops were consistent with Plastina et al*.*^56^ in the Midwest USA. Likewise, Bollero and Buck^57^ and Acuna and Villamil^33^ reported lower grain corn and soybean yield with CCs than without. Furthermore, when compensation was received for greater SOC storage with CC usage, profit margins for the CC treatments for grain and oilseed crops were no longer significantly less than those of the no-CC control. A payment of $50 C Mg^−1^ was needed to offset the costs of CCs in grain and oilseed crops. Innovations on how to enhance grain and oilseed crop yield gains in response to CCs is needed. Our results indicate that profitability from CCs was most dependent on the main crop grown (i.e. grain and oilseed crops vs. processing vegetable crops) followed by CC species; thus, future research regarding the crop-specific economic returns from CCs is needed.

It is worth acknowledging that the low profit margins in grain and oilseed crops was largely influenced by two out of four years of wheat. Profits in wheat are known to be low, which partially explains the decline in small grain acreage in Ontario and the Midwest USA^58^. Another contributing factor to profit analysis was the inclusion of the cost to control cereal rye. Herbicide application before planting the main crop is considered a typical farm management practice as many fields have weeds. Therefore, since herbicide is a typical business cost and not a cost generated by CC use, the results pertaining to the profitability of cereal rye and OSR + Rye would improve. It was necessary to include the cost of herbicide application in the profit analysis since some method of cereal rye termination is required and chemical control is the most common method of termination employed in temperate climate, conventional agriculture as well as what was used in this long-term experiment.

Our study results show no economic incentives to grain and oilseed producers to use CC regardless of C pricing. The potential loss in profits is a significant barrier to adoption and provides a partially explanation for the relatively low percent of (13.7%) of farmers that reported winter CC use^59^. Given that majority of Ontario cropland is under grain and oilseed production and a much greater proportion in the Midwest USA, the potential for wide-spread CC adoption appears limiting. Notwithstanding the SOC and associated soil quality gains from CCing, if the goal is to increase CC use for public environmental benefits (such as C sequestration, mitigating nutrient loading into water sources, and minimizing soil erosion) then government financial support may be necessary to incentivise CCs, particularly among grain and oilseed producers.

## Conclusions

This study indicates the positive influences of annual CCs in increasing C storage in surface soil after using CC 6 times over 8 years. Among the tested CCs, OSR contributed to greatest cumulative plant above-ground C inputs and SOC gains. Compared with the no-CC control, all CCs had greater SOC content and had as good as or better crop yields indicating the suitability of the tested CCs in improving soil functionality, primary productivity and sequestering atmospheric CO_2_ in our temperate humid climate. Farm-level profit margins in processing vegetable crops were greater for all CCs but oat (which was not different than no-CC control regardless of C pricing). It is evident from these results that profit margins for grain and oilseed producers are likely to decrease with CC use. This indicates the need for future research focusing on identifying and recommending the appropriate CC species for enhancing the profitability to the growers. The implementation of a policy that compensates producers for sequestering C may offset the losses in profits associated with CC usage and provide incentives for adoption. This was particularly found to be the case with a C price of $50 Mg^−1^. Accordingly, without additional financial compensation, grain and oilseed producers bear financial losses of CC usage. To our knowledge, this is the first research to quantify economic returns based on C pricing with the quantity of C sequestered in surface soil due to CCing. Future research is needed to better understand the CC-induced benefits on economics and environmental services.

## Materials and methods

### Experiment description and design

A long-term CC experiment was established in 2007 (site A) and repeated in 2008 at an adjacent site (site B, at a distance of 6 m) at University of Guelph, Ridgetown Campus, Ontario, Canada. The experiment has been previously described in detail in several studies^13–14,18–20,40^. Study site had a temperate humid climate with an evenly distributed average total monthly precipitation of 72.4 mm and mean annual air temperature of 9.6 °C from 1986 to 2016. Soil texture at both sites was a sandy loam (Orthic humic gleysol)^60^. Mean and standard error of the site soil characteristics measured in 2015 at site A were pH (6.12 ± 0.105), P (5.6 ± 0.499 mg kg^−1^), K (136 ± 7.38 mg kg^−1^) and 2016 at site B pH (7.06 ± 0.087), P (21.4 ± 4.98 mg kg^−1^), K (147 ± 14.8 mg kg^−1^). The initial soil organic matter at the experimental sites was 3.8% (measured by loss on ignition)^20^ but initial SOC content was not quantified. Crop rotation was identical at both sites and consisted of grain and processing vegetable crops followed by summer planted CCs in the sequence of pea-CC, sweet corn-CC, spring wheat-CC, tomato-CC, grain corn, squash-CC, soybean, winter wheat, and tomato-CC (see Table 1 for crop rotation). Depending on the main crop harvest date, CCs were either planted in July, August or September and remained in place until the following spring. Approximately three weeks after CC planting, glyphosate at 1 L ha^−1^ was applied, when necessary, to control weeds in the no-CC control plots. Cereal rye CC was terminated by spraying the entire trial area with glyphosate at 1.5 L ha^−1^ in the following spring season, whereas the remaining CCs were winter terminated. Fertilizer N, P, and K was applied to the main crop. See Chahal and Van Eerd^13–14^, Belfry et al*.*^18^, O’Reilly et al.^20^ for further production details. Main crops and CCs were managed in accordance with typical Ontario production practices^13,14,18,20,40^.

At both sites, experiment was arranged as a split-plot design with 4 replicates. Main plot (16 by 6 m) had five CC treatments arranged in a randomized complete block design (no cover crop control (no-CC), oat (*Avena sativa* L.), oilseed radish (OSR, *Raphanus sativus* L. var. *oleoferus* Metzg. Stokes), cereal rye (*Secale cereale* L.), and a mixture of OSR and cereal rye (OSR + Rye), which were drilled at 81, 67, 16, and 9 + 34 kg ha^−1^ year^−1^. While not of interest in this study of C sequestration and economics, the split-plot was either crop residue management (i.e., wheat and grain corn) or N fertilizer rate treatments (i.e., sweet corn and 1st-year tomatoes) and depended on the main crop grown. The experiment at both sites consisted of 20 main plots (5 CC treatment × 4 replications) and 40 split-plots (5 CC treatment × 4 replications × 2 crop residue or N fertilizer treatment).

### Crop and soil sampling

To quantify CC C content, biomass was sampled in the fall of every year, prior to freeze up (late October or early November), using two 0.25 m^2^ quadrats combined in each plot (i.e., main plot (n = 20 ) or split plot (n = 40) when treatment was applied). At harvest (usually in September or October depending on the crop), main crop yield (fruit or grain) was quantified and main crop aboveground biomass samples were collected from each plot except soybean and winter wheat, where main crop biomass was calculated using grain yield and a harvest index of 0.55 and 0.47, respectively. Biomass samples from CC and main crop were dried in a kiln at 60 °C, weighed, ground in a Wiley mill, and analyzed via combustion on LECO CN analyzer (Leco Corporation St. Joseph, MI) to determine the C concentration. Carbon content assimilated in above-ground biomass of CC and main crop was calculated annually from 2007 to 2015 (site A) and 2008 to 2016 (site B) by multiplying the above-ground dry biomass obtained in each split-plot by its C concentration. Cumulative plant C inputs over the nine years were calculated by adding the annual C inputs from each split-plot.

Soil was sampled from each split-plot (n = 40 at each site; 5 CC treatments × 2 crop residue treatments × 4 replications) to quantify SOC stock from 0–15 cm depth after tomato harvest in September 2015 and 2016 at both sites. From each split-plot, 20 random soil cores (3.5 cm diameter) were collected, combined, hand homogenized, and stored at 4 °C until sieved through 4 mm mesh screen. A 5 g soil sub-sample was oven-dried at 60 °C for 24 h^61^ to determine water content. A 0.15 g sub-sample was combusted (LECO CN analyzer) to quantify total C concentration on described above, while another 15 g soil sub-sample was combusted in a muffle furnace at 420 °C for 24 h before quantifying inorganic C on LECO CN analyzer. Soil organic C concentration was calculated as the difference between total and inorganic C. Soil organic C content was represented by the product of SOC concentration, soil bulk density (7.5 cm diameter ring, 7.5 cm in height), and depth of sampling and expressed as Mg C ha^−1^.

### Economic and statistical analysis

An initial statistical analysis of all data was conducted for a split-plot design where the fixed effects of sampling year (crop parameters only as SOC and economics were conducted once), CC (main plot effect), crop residue or N fertilizer management (split-plot effect), and their interactions were evaluated. Random effects were replication and replication by CC (to account for the split-plot effect). The results of the initial statistical analysis revealed no significant effect of split-plot treatment nor its interaction with CC or sampling year on any of the tested variables. Therefore, the analysis focused only on the main effects of CC treatments to assess the long-term cumulative impact of CCs on soil C storage and profit margins. Short-term impacts of main crop residue management and N fertilizer application (i.e., split-plot treatments) were not included in the economic and statistical analyses as these treatments had no significant effect. The database from which economic and statistical analyses were conducted contained all data (i.e., n = 40 for each site).

The economic analysis sought to identify differences in main crop yield and profit margins for each of the CC treatments, relative to the no-CC control. Crop yield data was derived from the experiment data. Yield data were also used to calculate profit margins over CC costs, which were calculated as the difference between revenues and additional costs associated with CCs (i.e., those that vary with management). Revenues were calculated as the product of each plot yield and the average market price for the year in which the crop was harvested, which was derived from OMAFRA’s statistics for field crops and for horticultural crops^62^. Costs associated with CCs included seed costs, planting costs, and herbicide product and application costs for the no-CC control (fall application) and for the CC treatments that included cereal rye (for termination in the spring). Seed costs ($45.15 to $83.03 ha^−1^) were based on prices provided by seed retailers in southern Ontario, costs of custom planting ($46.95 ha^−1^) and custom herbicide application ($22.24 ha^−1^) were based on OMAFRA’s 2015 Custom Rate Survey^63^, and the cost of glyphosate ($8.95 L^−1^) was derived from the Ontario Farm Input Monitoring Surveys (average of reported prices between 2008 and 2016). All other input costs, such as land costs and seed, fertilizer, and herbicide costs for the main crop, were assumed to be equal across treatments, and as such were not included in the calculation of profit margins.

Since yields and profit margins vary considerably among crops, combining and analyzing yield and profit margin data across all years and crops would not generate meaningful results. Instead, for the study duration of 2007–2015 at site A and 2008–2016 at site B, yield ratios and profit margin ratios were calculated between each CC treatment and the no-CC control from each year and for each crop to permit more appropriate comparisons among CCs across multiple crops and years of data. For each year and site, yield ratios for each crop were calculated by dividing the main (or split-plot, if present) plot yield with the average yield from corresponding no-CC controls (i.e., average of the 4 replicates; n = 4). The same approach was done to calculate profit margin ratios. A ratio equal to 1 represented no difference in yield (or profit margin) between the CC treatment and no-CC average in each year, whereas a ratio > 1 represented a gain in yield (profit margin) relative to the no-CC and a ratio < 1 represented a loss. For example, a yield ratio of 1.2 for OSR would indicate that the average yield for the OSR treatment in that year was 20% higher than the average yield with no-CC.

Regression analysis was conducted using Stata (StataCorp LLC, version 13, College Station, TX, USA) to examine for differences between CC treatments and the no-CC control in profit margin ratios. A mixed model regression approach was used to account for both fixed and random effects. The fixed effects included the CC treatments, for which categorical variables were specified for each of the four CC (for example, oat was set to 1 for all plots where oat was seeded as a CC and was set to 0 for all other plots). The random effects component of the model accounted for variation in profit margin ratios among the two sites and four blocks of the field experiment.

The initial regression analysis was conducted for data across all years and crops. Subsequently, to address potential differences in results arising from different types of crops, analysis of yield ratios and of profit margin ratios was conducted separately for grain and oilseed crops and for vegetable crops. The grain and oilseed crops included spring wheat, corn, soybean, and winter wheat, while the processing vegetable crops included sweet corn, tomato (planted twice), and squash.

Additional analysis was then conducted on profit margin ratios that took into account potential compensation for the amount of C sequestered in the soil due to the use of CCs. For this analysis, differences in the amount of SOC for the CC treatments, relative to the average amount of SOC for the no-CC control (n = 4), were calculated for each split-plot. These differences were multiplied by the price of C and added to the profit margins prior to the calculation of profit margin ratios. This analysis was conducted separately under two price scenarios for carbon: $20 tonne^−1^, the existing price in Canada at the time the analysis was conducted (2019), and $50 tonne^−1^, the price that was projected for 2022 under the Pan-Canadian Framework on Clean Growth and Climate Change^64^. This analysis was conducted for the combined data across all crops as well as separately for grain and oilseed crops and for processing vegetable crops. The purpose of this component of the analysis was to determine whether compensation based on Canada’s current C pricing policy would influence the results regarding the impacts of CC on profit margins. It should be recognized that although Canada has set C prices, there is currently (May 2020) no mechanism in place to compensate farmers (pers. comm. Mr. Don Lobb, farmer and Honorary Life Member of the Soil Conservation Council of Canada).

Crop (CC and main crop) and soil data were analyzed in SAS (SAS Institute, version 9.4 Cary, NC, USA), using PROC GLIMMIX with each site analyzed separately due to a significant interaction between CCs and sites at *P* < 0.05. To evaluate the CC effects on plant C inputs and SOC content from 0 to 15 cm depth, the statistical model in SAS, consisted of CC, sampling year, and interaction of CC and sampling year as fixed effects while replication was a random effect. All the assumptions of ANOVA were met as confirmed by the studentized residuals and normality test (Shapiro–Wilks)^65^. For all data, treatment means were compared using protected LSD test at a significance level of 0.05. Spearman correlation (using PROC CORR) was conducted to determine the relationship between fall CC above-ground C content and the subsequent crop yield for each year. Additionally, regression analysis, PROC REG, was used to assess the association between average crop yield ratio (or the variability between years) with SOC content at both sites (i.e., from 2008 to 2015 at site A and 2009 to 2016 at site B).

## Data Availability

All data generated or analysed during this study are included in this published article.
